# Secondary Modification of S100B Influences Anti Amyloid-β Aggregation Activity and Alzheimer’s Disease Pathology

**DOI:** 10.3390/ijms25031787

**Published:** 2024-02-01

**Authors:** Romina Coelho, Chiara A. De Benedictis, Ann Katrin Sauer, António J. Figueira, Hélio Faustino, Andreas M. Grabrucker, Cláudio M. Gomes

**Affiliations:** 1 BioISI—Instituto de Biosistemas e Ciências Integrativas, Faculdade de Ciências, Universidade de Lisboa, 1749-016 Lisboa, Portugal; rjccoelho@ciencias.ulisboa.pt (R.C.); ajfigueira@ciencias.ulisboa.pt (A.J.F.); 2 Departamento de Química e Bioquímica, Faculdade de Ciências, Universidade de Lisboa, 1749-016 Lisboa, Portugal; 3 Cellular Neurobiology and Neuro-Nanotechnology Laboratory, Department of Biological Sciences, University of Limerick, V94PH61 Limerick, Ireland; chiara.debenedictis@ul.ie (C.A.D.B.); ann.katrin.sauer@ul.ie (A.K.S.); 4 Bernal Institute, University of Limerick, V94PH61 Limerick, Ireland; 5 Health Research Institute (HRI), University of Limerick, V94PH61 Limerick, Ireland; 6 Research Institute for Medicines (iMed.ULisboa), Faculdade de Farmácia, Universidade de Lisboa, 1649-003 Lisboa, Portugal; heliofaustino@campus.ul.pt; 7 Association BLC3—Technology and Innovation Campus, Centre Bio R&D Unit, Oliveira do Hospital, Rua Nossa Senhora da Conceição No. 2, 3405-155 Coimbra, Portugal

**Keywords:** Alzheimer’s, neurodegeneration, S100 proteins, amyloid beta, neuroinflammation, oxidation, cytokine, molecular chaperones

## Abstract

Proteinaceous aggregates accumulate in neurodegenerative diseases such as Alzheimer’s Disease (AD), inducing cellular defense mechanisms and altering the redox status. S100 pro-inflammatory cytokines, particularly S100B, are activated during AD, but recent findings reveal an unconventional molecular chaperone role for S100B in hindering Aβ aggregation and toxicity. This suggests a potential protective role for S100B at the onset of Aβ proteotoxicity, occurring in a complex biochemical environment prone to oxidative damage. Herein, we report an investigation in which extracellular oxidative conditions are mimicked to test if the susceptibility of S100B to oxidation influences its protective activities. Resorting to mild oxidation of S100B, we observed methionine oxidation as inferred from mass spectrometry, but no cysteine-mediated crosslinking. Structural analysis showed that the folding, structure, and stability of oxidized S100B were not affected, and nor was its quaternary structure. However, studies on Aβ aggregation kinetics indicated that oxidized S100B was more effective in preventing aggregation, potentially linked to the oxidation of Met residues within the S100:Aβ binding cleft that favors interactions. Using a cell culture model to analyze the S100B functions in a highly oxidative milieu, as in AD, we observed that Aβ toxicity is rescued by the co-administration of oxidized S100B to a greater extent than by S100B. Additionally, results suggest a disrupted positive feedback loop involving S100B which is caused by its oxidation, leading to the downstream regulation of IL-17 and IFN-α2 expression as mediated by S100B.

## 1. Introduction

The impaired clearance of misfolded proteins during aging can result in neurotoxic aggregated proteins accumulating in the brain, leading to progressive neurodegeneration [1,2,3]. This includes protein inclusions inside neurons and in the extracellular space that affect cell-to-cell communication and health [4,5]. Inflammation is a biological response that is thought to occur downstream of these insults to the brain [6,7,8]. The activation of a number of cytokines and other molecules—the so-called alarmins [9]—results in an amplification of stress-related responses aimed at mitigating neuronal damage. Among these alarmins are the S100 proteins [10,11].

S100 proteins are a family of small regulatory calcium-binding proteins that act in a concentration-dependent fashion and have tissue and cell-specific expression [12]. Calcium binding via EF-hand motifs induce the conformational changes that trigger functional interactions with other proteins [13,14]. Furthermore, several S100 proteins have additional regulatory binding sites for zinc and copper [15]. At low concentrations (nanomolar), S100 proteins exert primarily intracellular functions, whereas upon astrocyte activation and at high concentrations (micromolar), they are secreted and act as extracellular cytokines via Receptor for Advanced Glycation End products (RAGE)-mediated signaling [16,17]. RAGE is an immunoglobulin-like cell surface receptor up-regulated in AD. It triggers the expression of pro-inflammatory cytokines [18]. S100 proteins show increased expression linked to several risk factors for AD, including aging [19] and abnormal trace metal homeostasis [20]. Although S100B and S100A9 are prone to undergo self-assembly under physiological conditions [15,21], recently published work shows that S100B directly modulates the aggregation of amyloid beta 42 (Aβ_42_) [22]. In line with this, the cell health of cultured neurons is less affected by exposure to Aβ_42_ plus Ca^2+^-bound S100B [22,23]. Thus, S100B acts as a chaperone, counteracting protein aggregation. However, how this chaperone activity is controlled on a molecular level is currently poorly understood.

In the presence of Aβ, astrocytes have been reported to generate oxidants. ROS-producing enzymes were found to be increasingly expressed and capable of signaling via the NF-kB transcription factor [24]. Therefore, S100B secreted by astrocytes will be exposed to an oxidizing milieu [25]. It is known that S100 protein oxidation can change S100 metal ion-binding properties [26], protein interactions [27], and extracellular functions [28,29]. Oxidized S100B likely occurs in the human brain [30]. Aβ-induced oxidative stress has additional direct and indirect implications to the pathophysiology of Alzheimer’s disease, while participating in processes that ultimately result in neurodegeneration [31]. These include the damage of neuron membranes, mitochondrial dysfunction, Ca^2+^ dysregulation, tau hyperphosphorylation, and the up-regulation of pro-inflammatory mediators [32]. In fact, some drugs and natural compounds were shown to exert neuroprotection against some of these AD hallmarks [33].

This study investigates the biological effects of oxidized S100B compared to non-oxidized S100B on Aβ aggregation, toxicity, and the potential to induce pro-inflammatory signaling via cytokine up-regulation in an astrocytic cell line. We hypothesize that S100B oxidation is a key process in controlling the activity of S100B and associated astrocytic feedback loops that mediate inflammation.

## 2. Results

### 2.1. Effects of Oxidized and Non-Oxidized S100B on Aβ_42_ Aggregation and Toxicity

**Figure 1:**
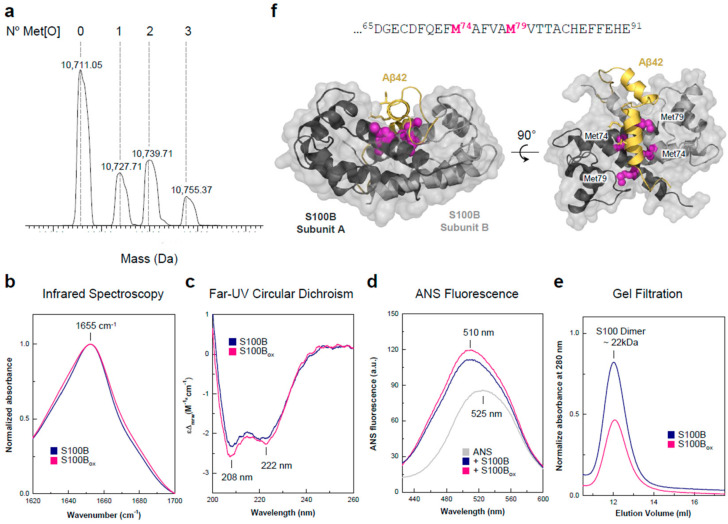
Characterization of S10S0B_ox_. (**a**) MS spectrum of S100B_ox_ depicting the peaks corresponding to the different degrees of methionine oxidation identified. S100B_ox_ structural characterization using (**b**) FTIR, (**c**) CD, (**d**) ANS fluorescence, and (**e**) size-exclusion chromatography. (**f**) Structural representation of a model of S100B (grey) bound to Aβ_42_ (yellow) generated in [40] highlighting S100B-Met residues (magenta) within the chaperone–client binding cleft, identified in [22].

To mimic S100B in vivo oxidation, we employed a mild oxidation protocol based on the HClO treatment [34] of recombinantly expressed S100B, herein designated as S100B_ox_. MS analysis of HClO-treated S100B reveals the occurrence of methionine oxidations, with peak masses compatible with the presence of up to three methionine sulfoxide residues per S100B_ox_ monomer (Figure 1a). Supporting such modifications, we also noted that the FTIR spectra of oxidized and non-oxidized S100B were distinct in the 1000–1100 cm^−1^ region in which vibrational modes associated with methionine sulfoxide have been identified [35]. MS analysis did not provide evidence for Cys oxidation, as confirmed by the reaction between S100B_ox_ and dansyl-maleimide [36], which introduced a 0.373 kDa mass shift per free sulfhydryl group. Biophysical characterization revealed that the structural properties of S100B are globally preserved upon oxidation (Figure 1b–e). In particular, the analyses of the secondary structure using attenuated total reflectance Fourier-transform infrared spectroscopy (ATR FTIR) and far-UV Circular Dichroism (Far-UV CD) show that S100B_ox_ retains the typical α-helical topology of S100 proteins, as evidenced, respectively, by the FTIR amide I band centered at 1655 cm^−1^ (Figure 1b) and by the negative CD bands at 208 and 222 nm (Figure 1c), which are typical of α-helices [37].

Similarly, the characteristic conformational change that occurs upon Ca^2+^ binding to S100 proteins and results in the exposure of a hydrophobic patch [14,38] was observed irrespective of the oxidation status of S100B, as inferred from the analysis of 8-Anilinonaphthalene-1-sulfonic acid (ANS) fluorescence. ANS is a hydrophobicity-sensitive fluorophore whose fluorescence emission increases and blue-shifts from 510 to 525 nm upon interaction with hydrophobic moieties following excitation at 370 nm [39]. Indeed, we observed that Ca^2+^ binding to apo S100B and S100B_ox_ resulted in identical ANS emission spectra. Finally, we used gel filtration analysis to determine if mild oxidation affected the S100B quaternary structure, and concluded that the treatment did not alter the dimeric state of S100B (Figure 1e). Overall, these results show that S100B_ox_ retains the folding and structure of the unmodified protein.

**Figure 2:**
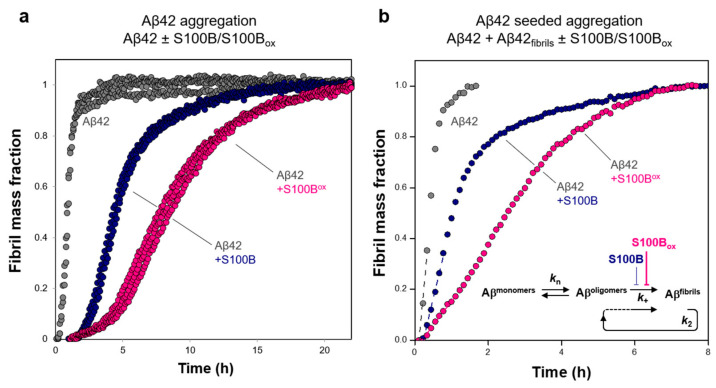
Effects of oxidized and non-oxidized S100B on Aβ_42_ aggregation. (**a**) Kinetic traces of ThT-monitored aggregation of monomeric Aβ_42_ (5 μM) in the absence and presence of a 10-fold excess ratio of S100B (blue) and S100B_ox_ (magenta) (in all cases *n* = 3). (**b**) Kinetic traces of ThT-monitored aggregation of monomeric Aβ_42_ (5 μM) in the absence and presence of a 4-fold excess ratio of S100B (blue) and S100B_ox_ (magenta) and seeded with 0.04 µM of pre-formed Aβ_42_ fibrils (average traces, *n* = 3).

Interestingly, structural analysis showed that methionines 74 (Met74) and 79 (Met79) were located within the S100B binding cleft responsible for the interaction with monomeric Aβ_42_ (Figure 1f), as previously identified by NMR and molecular dynamic simulations [22,40]. This binding cleft is located at the interface of the S100B homodimer, and comprises several ionizable and hydrophobic residues, including Met74 and Met79, that engage dynamic interactions with Aβ42 aggregation-prone segments [22]. Therefore, we hypothesize that oxidation of such methionine residues might modulate the anti-aggregation chaperone activity of S100B. To test this hypothesis, we investigated the effect of S100B_ox_ on the kinetics of Aβ_42_ aggregation compared to that of unmodified S100B using the well-established thioflavin (ThT) assay [22,41,42]. We observed that S100B_ox_ had a more substantial effect on delaying Aβ_42_ aggregation in comparison with non-modified S100B (Figure 2a), resulting in an increase in the aggregation half-time from 4.5 h (+S100B) to 8.5 h (+S100B_ox_) versus 0.9h for Aβ_42_ alone. Next, we compared the effect of S100B_ox_ versus that of S100B on Aβ_42_ fibril elongation using Aβ seeded aggregation assays (Figure 2b). Briefly, Aβ_42_ aggregation into fibrils proceeds through a series of microscopic states (see inset in panel Figure 2b) that involve the self-assembly of Aβ monomers (primary nucleation, at rate k_1_), with the formation of early fibrillar oligomers [43] that evolve to mature fibrils, upon the addition of Aβ monomers to the fibril ends (elongation phase, at rate k_+_) [44,45]. Interactions of the Aβ monomers with the fibrils prompt the formation of more oligomers (secondary nucleation, at rate k_2_) [46]. Experimentally, the influence of a chaperone (or any other aggregation modulator) on fibril elongation can be accessed by employing conditions that accelerate primary nucleation, which can be achieved by adding pre-formed Aβ fibrils that will seed aggregation [43,47]. In this case, aggregation assays in the presence of a high concentration of pre-formed Aβ_42_ fibrils were then employed to assess how S100B_ox_ affected fibril elongation. We noted that S100B_ox_ is about three times more efficient than S100B in blocking fibril elongation (Figure 2b).

### 2.2. Effects of Oxidized and Non-Oxidized S100B on Cell Heath in AD

**Figure 3:**
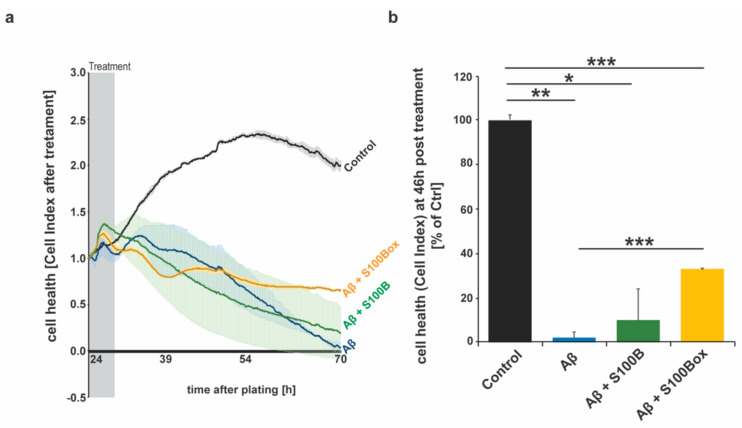
Cell health assessments using E–16 plates and an xCELLigence RTCA instrument. (**a**) Real-time impedance traces obtained using real-time monitoring of astrocyte cell adhesion and proliferation that had been treated with Aβ_42,_ Aβ_42_ + S100B, and Aβ_42_ + S100B_ox_ (*n* = 2–3 wells per condition). While in the first phase, no difference between the treatment conditions was observed, cells exposed to Aβ_42_ + S100B_ox_ showed recovery starting at around 15 h post-treatment. Untreated control cells proliferate until 100% confluency, and cell health decreases after reaching this point (54 h). After Aβ_42_ treatment, a continuous decrease in cell health was observed due to the detachment of dead cells. (**b**) After 46 h, significant differences between treatment conditions were observed (one-way ANOVA, F = 23.3995; *p* = 0.0023). Aβ_42_ treatment significantly reduced cell health compared to untreated controls (** *p* = 0.0013). Additional S100B treatment did not significantly reduce the toxicity of Aβ_42_ (Aβ_42_ vs. Aβ_42_ + S100B: *p* = 0.64; Ctrl_2_ vs. Aβ_42_ + S100B: * *p* = 0.0244). In contrast, S100B_ox_ partially rescued Aβ_42_ toxicity (Ctrl vs. Aβ_42_ + S100B_ox_: *** *p* = 0.0001; Aβ_42_ vs. Aβ_42_ + S100Box: *** *p* = 0.0005).

In the following experiments, we investigated whether S100B oxidation can modify Aβ_42_-induced toxicity using an astrocytic cell line. To that end, the health of DI TNC1 cells was assessed by measuring the impedance of astrocytes grown for 24 h (Figure 3a,b). In real-time, cell proliferation/cell viability was monitored after adding a low molecular species of Aβ_42_ (5 µM) [48,49], with or without S100B or oxidized S100B in a ratio of 1:2 for another 46 h. The results show that Aβ_42_ treatment induces evident cell toxicity. S100B co-administration did not result in a significantly lower Aβ_42_-induced toxicity. In contrast, co-treatment with S100B_ox_ led to significantly lower Aβ_42_-induced toxicity (Figure 3b) after 46 h of treatment.

### 2.3. Cytokine Gene Expression Is Modified by S100B Oxidation

The release of inflammatory cytokines has been reported in AD model systems as well as in patients [6,7]. For example, IL-17 levels are elevated in AD [50]. IL-17 (or IL-17A) is one of six members of the 17A-F cytokine family. Through the IL-17 receptor, IL-17 mediates activation of the transcription factor in NF-kB and downstream kinases. These kinases, in turn, lead to the generation of further pro-inflammatory signaling molecules and the attraction of immune cells to the site of inflammation [51]. Depleting IL-17 with an IL-17 antibody pre-treatment prevented Aβ_42_-induced neurodegeneration and memory decline in mice [52]. In addition, Interferon-alpha (IFN-α) is a cytokine found to be elevated in the frontal cortex of AD brains [53]. Astrocytes mainly express IFN-α upon Toll-like receptor activation. Interferons are helical cytokines, and, like IL-17, they mediate pro-inflammatory signaling [54].

**Figure 4:**
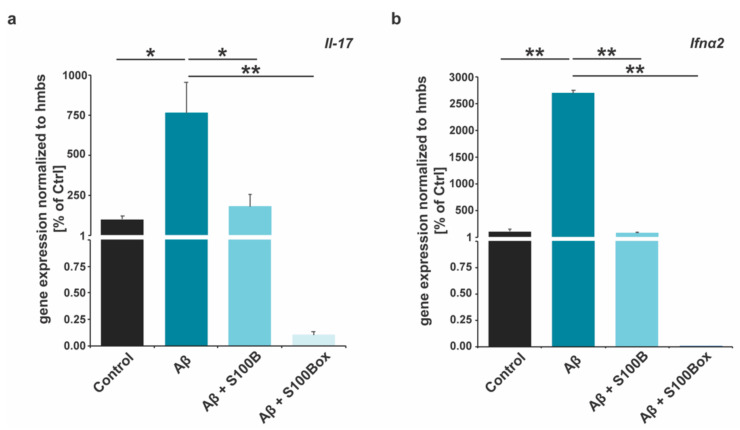
Inflammatory cytokine expression after exposure to Aβ_42_ with and without S100B or oxidized S100B. DI TNC1 astrocytes were treated with 5 µM Aβ_42_ and 10 µM S100B or S100B_ox_ plus Aβ_42_. RNA expression levels were normalized to *hmbs* and presented in the percentage of control (*n* = 3). (**a**) Significant changes were detected in *Il-17* expression (one-way ANOVA: F = 9.4102; *p* = 0.0075). Aβ_42_ treatment significantly increases *Il-17* expression (Tukey’s test, * *p* = 0.0136). The addition of S100B normalizes *Il-17* expression levels (Tukey’s test, Aβ vs. Aβ + S100, * *p* = 0.0252; Ctrl vs. Aβ + S100, n.s.). S100B_ox_ also down-regulates Aβ_42_-induced *Il-17* expression (Tukey’s test, Aβ vs. Aβ + S100_ox_, ** *p* = 0.0065; Ctrl vs. Aβ + S100_ox_, n.s.). (**b**) Significant changes were detected in *Ifn-α2* expression (one-way ANOVA: F = 989.4808, *p* = 0.0000034). Aβ_42_ treatment significantly increases *Ifn-α2* expression (Tukey’s test, ** *p* = 0.00101). The addition of S100B normalizes *Ifn-α2* expression levels (Tukey’s test, Aβ vs. Aβ + S100, ** *p* = 0.0010053; Ctrl vs. Aβ + S100, n.s.). S100B_ox_ also down-regulates Aβ_42_-induced *Ifn-α2* expression (Tukey’s test, Aβ vs. Aβ + S100_ox_, ** *p* = 0.0010053; Ctrl vs. Aβ + S100_ox_, n.s.).

Therefore, next, we evaluated whether S100B oxidation could modify Aβ_42_-induced cytokine release from astrocytes. To that end, we quantified the gene expression of IL-17 and IFN-α after the Aβ_42_ treatment of astrocytes in cultures with and without S100B and S100B_ox_. Our results show that IL-17 mRNA levels significantly increase after Aβ_42_ treatment compared to untreated controls (Figure 4a). Co-treatment with non-oxidized S100B reduces Il-17 to control levels and, thus, significantly lowers Aβ_42_-induced expression. Oxidized S100B_ox_ similarly leads to a significant rescue of Aβ_42_-induced IL-17 up-regulation. We observed comparable results when investigating IFN-α2 expression. IFN-α2 mRNA levels significantly increased after Aβ_42_ treatment. Co-treatment with non-oxidized S100B reduced IFN-α2 to control levels. S100B_ox_ also led to a significant rescue of Aβ_42_-induced IFN-α2 up-regulation (Figure 4b).

### 2.4. Autoregulation of S100B Gene Expression

**Figure 5:**
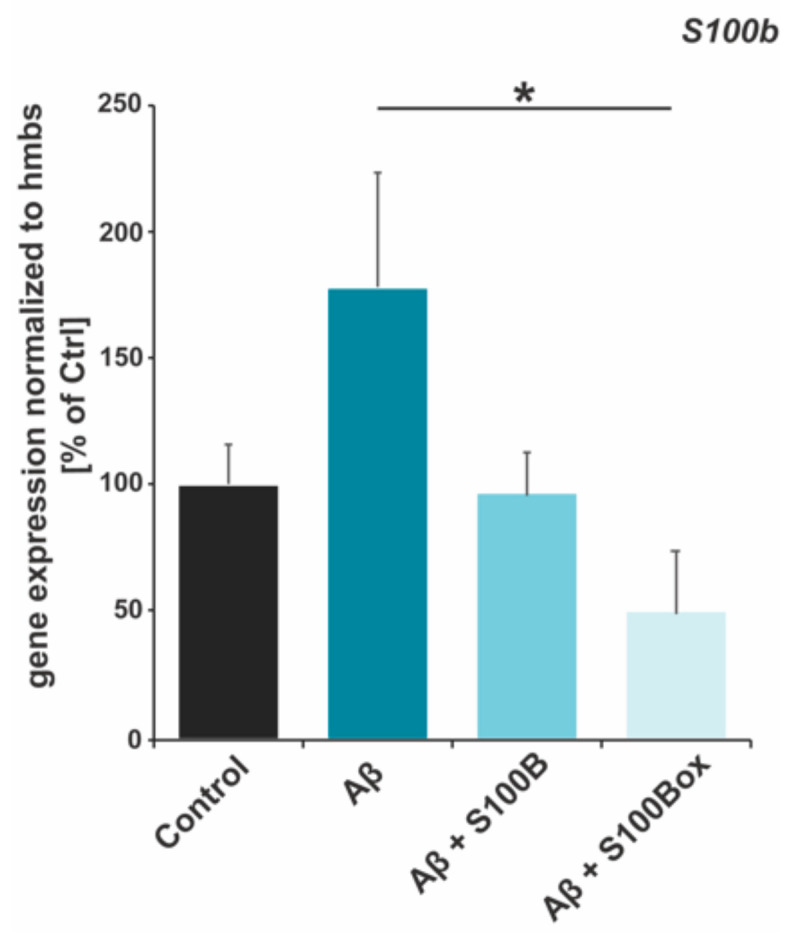
*S100b* gene expression after exposure to Aβ_42_ with and without S100B or oxidized S100B. DI TNC1 astrocytic cells were treated with Aβ_42_ and Aβ_42_ plus 4 µM S100B and oxidized S100B peptides (ratio 2:1). RNA expression levels were normalized to *hmbs* and are shown in percent of control (*n* = 3–6). Aβ_42_ treatment increases *S100b* expression. Only S100B_ox_ leads to a significant rescue (one-way ANOVA: F = 3.5366, *p* = 0.05, with Tukey’s test: Aβ vs. Aβ + S100_ox_, * *p* = 0.0395671; Ctrl vs. Aβ + S100_ox_, n.s.).

Astrocyte activation led to the increased expression of *S100b*, which has also been reported in AD [31]. This increase can also be modeled in astrocyte cultures (Figure 5), where we detected an *S100b* gene expression increase after Aβ_42_ treatment. The current hypothesis is that, under mild proteotoxic stress, S100B also acts as a chaperone, normalizing insults by inhibiting Aβ aggregation [22,55,56] and chelating excessive trace metals such as zinc and copper [57,58,59]. Therefore, the levels of S100B need to be tightly controlled. One possibility is that S100B oxidation levels could indicate a need for S100B and, thus, a control of S100B expression. To investigate this, *S100b* mRNA levels were determined after Aβ_42_ treatment in the presence of S100B or S100B_ox_ (Figure 3). Our results show that S100B_ox_- but not S100B-treated astrocytes had a significantly reduced Aβ_42_-induced expression of *S100b*.

## 3. Discussion

Molecular chaperones play key roles in proteostasis regulation, preventing misfolding and the accumulation of toxic aggregates which are a hallmark in several age-related neurodegenerative diseases [60,61]. Molecular chaperones are potentially neuroprotective, given their ability to modulate initial aberrant protein interactions that prevent toxic conformers that trigger pathogenic cascades [62]. This is commonly achieved by establishing protective interactions between a chaperone and its client that prevent or recover a misfolded conformed. One such holdase-type activity has been recently uncovered for S100B which is able to mitigate the pathological self-assembly of Aβ42 and to decrease toxicity [22,55]. This novel activity is particularly interesting, given the fact that S100B has both an intra- and extra-cellular function [63], being found to be associated with amyloid plaques in AD animal models upon their expression and secretion by activated astrocytes. This suggests its potential relevance in the biological setting and potential as a drug target, given the limited number of known secreted chaperones [64]. Importantly, the fact that S100B, like DNAJB6 [65,66] and the Brichos domain [67,68,69], are able to inhibit Aβ42 secondary nucleation, which is the main route to generate toxic oligomers, makes this type of proteins attractive inspirations for translational AD therapies [70,71,72].

The present study shows that S100B oxidation plays a critical physiological function in regulating S100B-mediated effects in AD. We show that oxidation of S00B more effectively diminishes the effect of Aβ on cells, and S100B_ox_ diminishes S100B transcription. In line with this, the AD astrocyte model reveals that S100B-dependent pro-inflammatory signaling (via IL-17 and IFN-α2 induction) is interrupted upon S100B oxidation. The results suggest a unique way of modifying the S100B function using oxidation that may occur in response to specific pathologies: through oxidation, the pro-inflammatory activity of S100B may be turned into an anti-inflammatory activity during the ‘Aβ_42_ detoxification’ by S100B, and, through oxidation, its own expression levels may be controlled.

This is supported by the finding that S100B rescues Aβ_42_ toxicity more effectively than S100B in an AD cell model. In part, this may be based on its reported chaperone activity. S100B may bind Aβ_42_ in the medium. In line with this, S100B_ox_ is indeed more effective in inhibiting the kinetics of Aβ_42_ aggregation. However, S100B could also induce astrocyte proliferation, which may contribute to an increase in the cell number and cell index. Low doses of S100B have been shown to stimulate astrocytic cell line proliferation [73].

Both S100B and oxidized S100B reduced inflammatory marker expression (IL-17, IFN-α2). IL-17A can induce pro-inflammatory gene expression via NF-kB, a known downstream target of S100B signaling [74]. DAMPS, such as S100B, activate several pathways leading to the activation of IRF kinases. Upon phosphorylation, IRF3 and IRF7 translocate to the nucleus and activate IFN-α2 transcription, among others [75]. Thus, our data suggest that IL-17 and IFN-α2 lie downstream of S100B signaling, but their induction may be sensitive to S100B oxidation. The differences between S100B and S100B_ox_ reported here may be an understatement, given that in the presence of Aβ_42_, some S100B will also be oxidized.

The significantly lower *S100b* mRNA levels of Aβ_42_ plus S100B_ox_ suggest that *S100b* gene expression might be particularly responsive to the presence of oxidized S100B. Subsequent down-regulation of IL-17 and IFN-α2 shows an anti-inflammatory function of S100B_ox_ and possibly suggests that S100B can down-regulate pro-inflammatory cytokines that are dependent on its oxidation. Under normal conditions, S100B up-regulates its gene expression in a positive feedback loop. However, this feedback loop may get, and need to be, interrupted, which may be mediated by S100B oxidation. Lin et al. (2010) report proof of a positive S100B feedback loop in cancer cells [76]. Following these results, S100B up-regulates its expression through a p53 binding. The interaction of S100B with p53 leads to an inhibition of p53, resulting in increased S100B expression in a negative feedback loop for p53 [76]. Interestingly, p53 has been linked to neurodegenerative diseases, including AD [77]. Moreover, S100B oxidation can modulate the interaction of S100B and the tumor suppressor p53 [16].

Taken together, the shift from S100B towards oxidized S100B could represent a shift in the balance between the pro-inflammatory and anti-inflammatory mechanisms, which may be a key factor in regulating S100B’s double-edged role in neuroinflammation in many conditions associated with increased S100B release.

## 4. Materials and Methods

### 4.1. Recombinant Protein Generation

Human S100B was expressed in *E. coli* cells, and dimeric S100B was purified following a previously established protocol [78]. To obtain apo S100B, S100B holo was incubated with a 300-fold excess of dithiothreitol (DTT) and 0.5 mM EDTA for 2 h at 37 °C. Afterward, it was injected and eluted in a Superdex S75 column (GE Healthcare, Chicago, IL, USA). To prevent trace metal binding, solutions were prepared with water passed through a Chelex resin (Bio-Rad, Hercules, CA, USA). Oxidation of S100B was carried out in a manner similar to a previously described protocol [34]. Hypochlorite (49.08 µL, 1.913 mmol) was added to S100B (4.3 mg, 201 nmol) diluted in 500 mM Tris-HCl pH7.4, incubated for 10 min at room temperature, and centrifuged for 5 min at 12,400 rpm, before being eluted in an S75 column (GE Healthcare). The S100B_ox_ protein was concentrated and stored at −20 °C. Human recombinant Aβ_42_ was expressed and purified as previously described [79]. The Aβ_42_ expression plasmid was kindly gifted by J.Presto (Karolinska Institutet, Solna, Sweden). Aβ_42_ expressing *E. coli* cells were harvested after 4 h by centrifugation and resuspended in 20 mM Tris-HCl pH8.0. For purification, Aβ_42_ cells were lysed by sonication. A centrifugation step was followed at 14,000 rpm for 20 min to isolate inclusion bodies. Next, the pellet was dissolved, sonicated for 3 min, 65 amplitude in a cycle of 0.5, and centrifuged again at 14,000 rpm for 20 min. Urea-solubilized inclusion bodies were then purified by anion-exchange chromatography and centrifugal filtration using a DEAE-cellulose column (GE Healthcare). Fractions containing solubilized Aβ42 were lyophilized and stored at −20 °C in low-binding tubes (Axygen Scientific, Corning, NY, USA). To obtain the low molecular species of Aβ_42_, 2.48 mg of lyophilized Aβ_42_ was dissolved in 1.7 mL 100% DMSO. For the lyophilized Aβ_42_ to dissolve, it was vortexed for 30 min, at a speed of 8, and water bath sonicated (pulses) at a frequency of 37 kHz for 3 min. The supernatant was kept following centrifugation at 8000 rpm. The concentration was determined by Nanodrop, and the Aβ_42_ stock was then stored at −20 °C. To obtain monomeric Aβ_42_ for ThT aggregation assays, about 2 mg was dissolved in 7 M guanidine hydrochloride and eluted in a Superdex S75 (GE Healthcare) with 50 mM HEPES (4-(2-hydroxyethyl)-1-piperazineethanesulfonic acid, NZYtech, Lisbon, Portugal) pH 7.4.

### 4.2. Mass Spectrometry

Intact protein analysis: The liquid chromatography–mass spectrometry (LC-MS) runs were realized using a Dionex Ultimate 3000 UHPLC+ system equipped with a Multiple-Wavelength detector, an imChem Surf BIO C4 300 Å 3 µm 150 × 2.1 mm column connected to Thermo Scientific Q Exactive hybrid quadrupole-Orbitrap mass spectrometer (Thermo Scientific™ Q Exactive™ Plus, Waltham, MA, USA). The mobile phase consisted of water with 0.1% FA (mobile phase A) and acetonitrile with 0.1% FA (mobile phase B). The following gradients were applied at a flow rate of 200 µL/min: precondition with 10% B for 5 min, linear gradient from 10% B to 80% B in 20 min. The electrospray source was operated with a Spray Voltage (+): 3.5 kV, Capillary Temperature 320 °C, Sheath Gas 47.50 (a.u.), Aux Gas 11.25. Deconvolution of peaks was performed using MagTran1.03 [80].

Digestion procedure with trypsin: 90 µL of NH_4_HCO_3_ (16 mg/mL), 18 µL TCEP solution (16 mg/mL in 30 mg/mL of NH_4_HCO_3_), and 72 µL of protein solution (4–6 µM) were combined and incubated at 60 ºC for 60 min. After cooling to room temperature, 21 µL of activated trypsin (0.1 µg/µL in NH4HCO3 (16 mg/mL) solution) was added and incubated at 37 °C for 16 h. The digestion was quenched with 15 µL of formic acid, vortexed briefly, and then centrifuged. Then, 20 µL of the solution was injected for MS analysis. Digestion procedure with formic acid: 100 µL of protein solution (100 µM) was added to 2 µL of formic acid and 3 µL of acetonitrile. The solution was incubated at 108 °C for 6 h, allowed to cool to room temperature, and then analyzed by LCMS. Analysis of digested peptides: The Liquid chromatography–mass spectrometry (LC-MS) runs were realized using a Dionex Ultimate 3000 UHPLC+ system equipped with a Multiple-Wavelength detector, an imChem Surf C18 TriF 100 A 3 µm 100 × 2.1 mm column connected to Thermo Scientific Q Exactive hybrid quadrupole-Orbitrap mass spectrometer (Thermo ScientificTM Q ExactiveTM Plus). Tryptic peptides were separated with a 0.2 mL/min and a mixture of water with 0.1% formic acid (buffer A), and acetonitrile with 0.1% formic acid (buffer B), using a gradient mixture of A:B solvents from 97:3 until 5:95 during 90 min. The Exactive mass spectrometer was operated in positive ion mode with alternating MS scans of the precursor ions and AIF (all ion fragmentation) scans in which the peptides were fragmented by HCD. Both scan types were performed with 100,000 resolution (at *m*/*z* 200), with each scan taking 1 s, and the maximal fill time was set to 1 s. The *m*/*z* range for MS scans was 300–1600, and the *m*/*z* range for AIF scans was 150–1600. The target value for the MS scans was 10^6^ ions, and the target value for the AIF scans was 3 × 10^6^ ions. HCD collision energy was 50 eV. A database search of possible modifications of digested peptides was performed using Skyline (64-bit) 20.2.0.343. The reaction of S100B and S100Box with maleimide: To a solution of S100B (106 µM approx.) (5 µL, 0.00053 µmol) or S100Box (151 µM approx.) (5 µL, 0.00076 µmol) in ammonium acetate at 20 mM pH 7 (106 µL) dansyl maleimide [30] was added at 10 mM in ACN (1.060 µL, 0.0106 µmol) and allowed to react for 4 h.

### 4.3. Aggregation Kinetics

Aβ_42_ aggregation kinetics were performed by recording Thioflavin-T (ThT) fluorescence intensity as a function of time in a plate reader (FLUOstar Optima, BMG Labtech, Ortenberg, Germany) with a 440 nm excitation filter and a 480 nm emission filter. The fluorescence was measured using the bottom optics in half-area 96-well polyethylene glycol-coated black polystyrene plates with a clear bottom (Corning, 3881, Corning, NY, USA). The microplates were sealed with foil to avoid evaporation. Monomeric Aβ_42_ was diluted to a final concentration of 5 µM in 50 mM of HEPES pH 7.4 and the indicated concentrations of S100B and S100B_ox_. Then, 10 μM of ThT (Sigma, St. Louis, MO, USA) was added to each condition. All assays were performed at 37 °C, under quiescent conditions, with fluorescence measurements taken every 400 s and with three technical replicates (*n* = 3).

### 4.4. ANS Fluorescence

Fluorescence measurements were performed on a Jasco FP8200 spectrofluorometer (Jasco, Tokyo, Japan). The temperature was kept at 25 °C by a Peltier-controlled cell support. For ANS analysis, S100B and S100B_ox_ were incubated with 2 molar equivalents of ANS for 30 min. ANS emission spectra were recorded using 10 nm excitation and emission slits upon 370 nm excitation.

### 4.5. Analytical Size-Exclusion Chromatography

Analytical size-exclusion chromatography was performed at room temperature on a Superdex 75 Tricorn high-performance column (GE Healthcare, bed volume = 24 mL) eluted at 1 mL/min, with 50 mM TrisHCl pH 7.4 using imidazole as internal standard.

### 4.6. Circular Dichroism (CD)

Circular dichroism (CD) measurements were performed on a Jasco J-1500 spectropolarimeter equipped with a Peltier-controlled thermostated cell support at 25 °C. Samples were prepared by diluting S100B to a final concentration of 5 µM (dimer equivalents) in 50 mM Tris-HCl pH 7.4. Far UV-CD spectra were recorded between 200 nm and 260 nm using a 1 mm pathlength quartz cuvette (Hellma Analytics, Müllheim, Germany) and a minimum of 8 scans of average accumulation.

### 4.7. Fourier-Transformed Infrared Spectroscopy (FTIR)

FTIR measurements were performed on a Bruker Tensor II FTIR Spectrometer (Billerica, MA, USA) equipped with a nitrogen-cooled MCT detector and a thermostatized Harrick BioATR cell at 25 °C. Before spectra acquisition, 20 µL of untreated and oxidized apo-S100B (≈250 µM) in 50 mM Tris-HCl pH 7.4 were pipetted into the ATR cell and equilibrated for 5 min. FTIR spectra between 900 and 4000 cm^−1^ were acquired with 120 technical accumulations, 12 mm of aperture, 20 kHz scanner velocity, 4 cm^−1^ spectral resolution, and buffer background correction.

### 4.8. Cell Culture

DI TNC1 (ATCC, Manassas, VA, USA) rat astrocytes were cultivated in Dulbecco’s modified Eagle medium (DMEM) containing 10% fetal bovine serum, 2% glutamine, 1% sodium pyruvate, and 1% penicillin–streptomycin. Cultures were maintained at 37 °C with 5% CO_2_ until they reached the 80% confluence necessary to perform the experiments.

### 4.9. Quantitative Real-Time PCR (qRT-PCR)

DI TNC1 cells were seeded on poly-L-Lysine (PLL) (0.1 mg/mL) coated Petri dishes of 10 cm in diameter and stored in the incubator until a monolayer (80% confluency) was reached. Afterward, the medium was replaced with a new medium for control or one already containing the treatment. The treatment of the Aβ_42_ condition was 5 µM Aβ_42_. For the S100B/ox plus Aβ_42_ condition, a co-administration of 10 µM S100B or oxidized S100B occurred, respectively. Then, 72 h later, total RNA isolation was performed using the RNeasy Mini kit (Qiagen, Manchester, UK), as described by the manufacturer. Isolated RNA was eluted in 30 µL RNAse-free water and kept at −80 °C. Quantitative real-time PCR was performed using the Rotor-Gene SYBR Green RT-PCR kit (Qiagen). First-strand synthesis and real-time qRT-PCR amplification (Roche LightCycler 480 II, Basel, Switzerland) was performed in a one-step, single-tube format. Validated primer pairs from Qiagen (Quantitect primer assay) were used. For the internal standard, obtained data were analyzed using the hydroxymethylbilane synthase (*hmbs*) gene. All reactions were run at least in technical triplicates. Virtual mRNA levels were calculated from mean ct values according to virtual mRNA level = 10 ((ct(target) − ct(standard))/slope of the standard curve.

### 4.10. Impedance-Based Cell Health Assay

DI TNC1 cells were seeded at 5000 cells per well on a PLL-coated E-16 plate (Agilent/ACEA Biosciences, San Diego, CA, USA). After attachment and 24 h of growth, the cells were treated with 5 µM Aβ_42_ or 5 µM Aβ_42_ co-administered with 10 µM S100B/10 µM S100Box. Controls were left untreated. Impedance was measured every 5 min in the following 70 h, employing the xCELLigence RTCA Systems (Agilent/ACEA Biosciences) with RTCA Software Pro Basic (https://www.agilent.com/en/product/cell-analysis/real-time-cell-analysis/rtca-software/rtca-software-pro-741236, accessed on 9 August 2022). A decrease in microelectronic impedance measured as (delta) cell index indicated a decrease in proliferation and detachment of the cells from the bottom of the wells to be a sign of cytotoxicity.

### 4.11. Statistical Analysis

For statistical analysis of the data sets, GraphPad Prism 8.0.2 was employed. All data are shown with standard error of the mean (SEM). Data were analyzed by one-way ANOVA, followed by appropriate post hoc tests (e.g., Tukey’s test). Statistical significance corresponded to a significance level of α ≤ 0.05. Significances are stated with *p* values < 0.05 *; <0.01 **; <0.001 ***.

## Data Availability

Data are available upon reasonable request.
